# Statement of Retraction: MicroRNA-624-mediated ARRDC3/YAP/HIF1α axis enhances esophageal squamous cell carcinoma cell resistance to cisplatin and paclitaxel

**DOI:** 10.1080/21655979.2024.2302646

**Published:** 2024-02-25

We, the Editor and Publisher of *Bioengineered*, have retracted the following article:

Jie Yan, Litong Shi, Shan Lin & Yi Li (2021) MicroRNA-624-mediated ARRDC3/YAP/HIF1α axis enhances esophageal squamous cell carcinoma cell resistance to cisplatin and paclitaxel, Bioengineered, 12:1, 5334-5347, DOI: 10.1080/21655979.2021.1938497

Following publication, the authors reached out to inform the journal in 2023 that they could not verify the data within the article. They stated that some of the experiments within the article were assisted by a third party and that the reported results could no longer be relied upon. The use of a third party to perform experiments was not disclosed within the article.

As we cannot verify the validity of the published work or that it complied with our editorial policies, we are therefore retracting the article. The authors listed in this publication have been informed.

 

We have been informed in our decision-making by our editorial policies and the COPE guidelines.

The retracted article will remain online to maintain the scholarly record, but it will be digitally watermarked on each page as ‘Retracted’. 

